# Patient Perspectives on Artificial Intelligence in Health Care: Focus Group Study for Diagnostic Communication and Tool Implementation

**DOI:** 10.2196/69564

**Published:** 2025-07-24

**Authors:** Garrett Foresman, Joshua Biro, Alberta Tran, Kate MacRae, Sadaf Kazi, Laura Schubel, Adam Visconti, William Gallagher, Kelly M Smith, Traber Giardina, Helen Haskell, Kristen Miller

**Affiliations:** 1 National Center for Human Factors in Healthcare, MedStar Health Research Institute, Washington, DC, United States; 2 Institute for Quality and Safety, MedStar Health, Columbia, MD, United States; 3 School of Medicine, Georgetown University, 3700 O St NW, Washington, DC, 20057, United States, 1 5745276685; 4 Center for Diagnostic Systems Safety, MedStar Health Research Institute, MedStar Health, Columbia, MD, United States; 5 University of Toronto, Toronto, ON, Canada; 6 Toronto East General Hospital, Toronto, ON, Canada; 7 Center for Innovations in Quality, Effectiveness and Safety, Michael E. DeBakey VA Medical Center, Houston, United States; 8 Section of Health Services Research, Baylor College of Medicine, Houston, United States; 9 Mothers Against Medical Error, Columbia, SC, United States

**Keywords:** co-design, diagnostic safety, artificial intelligence, patient engagement, communication

## Abstract

**Background:**

Artificial intelligence (AI) is rapidly transforming health care, offering potential benefits in diagnosis, treatment, and workflow efficiency. However, limited research explores patient perspectives on AI, especially in its role in diagnosis and communication. This study examines patient perceptions of various AI applications, focusing on the diagnostic process and communication.

**Objective:**

This study aimed to examine patient perspectives on AI use in health care, particularly in diagnostic processes and communication, identifying key concerns, expectations, and opportunities to guide the development and implementation of AI tools.

**Methods:**

This study used a qualitative focus group methodology with co-design principles to explore patient and family member perspectives on AI in clinical practice. A single 2-hour session was conducted with 17 adult participants. The session included interactive activities and breakout sessions focused on five specific AI scenarios relevant to diagnosis and communication: (1) portal messaging, (2) radiology review, (3) digital scribe, (4) virtual human, and (5) decision support. The session was audio-recorded and transcribed, with facilitator notes and demographic questionnaires collected. Data were analyzed using inductive thematic analysis by 2 independent researchers (GF and JB), with discrepancies resolved via consensus.

**Results:**

Participants reported varying comfort levels with AI applications contingent on the level of patient interaction, with digital scribe (average 4.24, range 2-5) and radiology review (average 4.00, range 2-5) being the highest, and virtual human (average 1.68, range 1-4) being the lowest. In total, five cross-cutting themes emerged: (1) validation (concerns about model reliability), (2) usability (impact on diagnostic processes), (3) transparency (expectations for disclosing AI usage), (4) opportunities (potential for AI to improve care), and (5) privacy (concerns about data security). Participants valued the co-design session and felt they had a significant say in the discussions.

**Conclusions:**

This study highlights the importance of incorporating patient perspectives in the design and implementation of AI tools in health care. Transparency, human oversight, clear communication, and data privacy are crucial for patient trust and acceptance of AI in diagnostic processes. These findings inform strategies for individual clinicians, health care organizations, and policy makers to ensure responsible and patient-centered AI deployment in health care.

## Introduction

Artificial intelligence (AI) has been widely adopted in numerous social and scientific areas, including integration into various health care applications [1,2]. AI offers great potential for improving patient care, especially in enhancing the early detection of diseases, automating routine works and tasks to manage patients and resources, and improving and streamlining workflow processes [3]. Through its ability to accommodate complex data, AI has shown great promise in enhancing diagnostic opportunities in a variety of clinical processes of the electronic health record, including diagnostic imaging, clinical decision support systems, and patient monitoring tools [4-8]. As AI continues to be rapidly deployed in various aspects of clinical practice, there are diagnostic safety implications given that diagnostic errors remain the leading cause of adverse outcomes in health care [9].

Existing research highlights the potential of AI to enhance diagnostic safety by identifying patterns in clinical data, improving early detection of diseases, and supporting decision-making [10-12]. For example, AI-based radiology tools have demonstrated the ability to detect pathologies like fractures or cancers with accuracy comparable to human radiologists [13,14]. Similarly, decision support tools use patient data to recommend diagnoses or tests, potentially mitigating diagnostic errors [15]. These AI tools, designed to support the diagnostic process, have the potential to reduce diagnostic errors by providing additional diagnostic information but can only do so through effective communication to ensure that patients are informed, engaged, and empowered in their care. As the adoption of these novel AI technologies will directly impact diagnosis and subsequently patient health outcomes, incorporating patient viewpoints into the design and implementation processes is critical to their widespread acceptance [16].

Few studies explore patient perspectives on AI’s role, particularly in diagnosis and diagnostic communication [17]. Including these perspectives is critical, as patient and family input can shape the development and use of AI in health care in ways that align with their needs, priorities, and values. Despite the rapid expansion of AI technologies, most studies to date focus on consumer attitudes toward AI broadly, emphasizing trust, acceptance, or perceived accuracy, rather than investigating how patients and families believe AI should be integrated into the diagnostic process and its communication [18-21]. These studies provide limited insights into the potential for AI to enhance shared decision-making, improve transparency in diagnostic reasoning, or address systemic inequities in health care delivery. Some research has examined patient attitudes toward AI in specific contexts, such as radiology. For instance, 2 recent studies explored patient perceptions of AI-based diagnostics in radiology image interpretation and the communication of results [22,23]. These studies highlight important themes, such as patients’ reliance on clinician expertise to contextualize AI findings and their concerns about how AI might influence trust in the diagnostic process. However, these findings remain confined to radiology and do not address broader questions about how AI can enhance diagnostic communication across diverse health care settings.

Furthermore, the role of families in understanding and interpreting AI-driven diagnostic information has been largely overlooked, despite evidence suggesting that family engagement can significantly impact health care outcomes [24,25]. This gap underscores the need for a more comprehensive exploration of how AI can support patient and family-centered care, particularly in fostering understanding, trust, and collaborative decision-making.

Our study aims to address this gap by investigating patient and family perspectives on AI’s role in diagnosis and diagnostic communication across various theoretical and practical applications through a co-design approach. By engaging patients and families as partners in this research, we seek to uncover their expectations, concerns, and preferences for how AI should be used to enhance diagnostic safety, support clinician-patient communication, and promote equitable health care delivery. This inquiry not only expands the existing body of literature but also provides actionable insights to inform the design and implementation of patient-centered AI technologies in health care.

## Methods

### Study Design and Setting

This study employed a focus group methodology informed by co-design principles to explore patient and family member perspectives on the use of AI in clinical practice. Focus groups were selected to facilitate rich, interactive discussions, enabling participants to build on each other’s insights while generating diverse perspectives. While the primary aim was to gather feedback on specific case scenarios illustrating AI use in health care, the session was structured to go beyond simple elicitation of opinions. Co-design principles were incorporated to actively engage participants in collaboratively identifying key concerns, priorities, and desired safeguards for AI integration. Rather than developing a tangible product, the co-design focus centered on shaping participant-driven guidelines and recommendations for how AI should be implemented in ways that support patient-centered care.

A single 2-hour session was conducted in a centrally located research office with a large conference room and breakout rooms. The session combined full-group discussions with smaller, scenario-specific activities designed to promote collaboration and iterative refinement of ideas. This approach allowed participants to reflect on real-world examples while contributing to the development of contextually relevant strategies for responsible AI use in clinical settings.

### Participants

Adult patients and family members aged 18‐80 years were recruited through email outreach, word of mouth, and networks such as our Patient and Family Advisory Council for Quality and Safety and the Georgetown University network. Recruitment focused on ensuring diversity in participant demographics, backgrounds, and health care experiences to capture a wide range of perspectives. Eligibility criteria included being English-speaking and willing to engage actively in the session.

### Procedures

The session was facilitated by a multidisciplinary team with expertise in human factors engineering, diagnostic safety, and patient engagement. Lead facilitators, trained in qualitative research methods, guided the session using a structured agenda designed to balance education, discussion, and cocreation. Recognizing the varying levels of participant familiarity with AI, the session began with an activity explicitly designed to surface and build a foundational understanding. This included group discussion of everyday AI examples (eg, navigation apps and virtual assistants) to ensure shared baseline knowledge before exploring health care–specific applications. We did not formally assess or quantify participants’ previous knowledge of AI or their digital literacy.

The session consisted of the following five key activities: (1) introduction and icebreaker (a brief overview of the session’s objectives, followed by an icebreaker to create a comfortable and engaging atmosphere), (2) “What is AI?” activity (participants discussed examples of AI in everyday life to build foundational understanding), (3) breakout sessions (participants were divided into smaller groups for 5 specific AI scenarios), (4) guideline or recommendation development, and (5) reflection and feedback (the session concluded with a debrief where participants shared insights and reflections). Materials provided to participants included an agenda, activity materials, and data collection for demographics and session evaluation.

In total, 5 scenarios were selected and developed to represent a diverse range of AI applications relevant to the diagnostic process and communication. These scenarios were designed to align with the study’s goal of examining patient perceptions of AI in diagnostic care by highlighting applications that varied in complexity, patient interaction, and clinical context. Each scenario was informed by a review of current AI use cases in health care and refined with input from our research team, including experts in diagnostic safety, human factors, and patient engagement. The five scenarios presented included: (1) portal messages (use of AI for patient portal messaging), (2) radiology review (use of AI in radiological imaging review), (3) digital scribe (ambient digital scribe for documentation in primary care), (4) virtual human (a virtual human presents a new diagnosis during a telehealth encounter), and (5) decision support (use of AI for clinical decision support to identify patients that would benefit from HIV testing) (Table 1). The scenarios were designed to elicit feedback based on different levels of patient interaction ranging from high interaction (virtual human and portal messages) where patients directly communicate with AI to indirect interaction (digital scribe) where AI is present during interaction with a human physician to minimal interaction (decision support and radiology review) where there is no direct communication between the AI and the patient. The specific scenarios can be found in Multimedia Appendix 1.

**Table 1.** Brief description of each scenario used for the co-design session.

Scenario	Description
Digital scribe	Before a routine checkup, the doctor asks permission to use an AI^a^-based app on their phone as a digital scribe to listen and document notes based on the visit.
Radiology review	A radiologist initially sees nothing on a CT^b^ scan for severe back pain, but AI software identifies a herniated disc, which the radiologist then confirms.
Decision support	During a routine wellness visit, an AI system recommends HIV screening based on interpreted medical and social history, prompting the clinician to offer the test.
Portal messages	After a routine visit with recommended laboratory work, a patient accesses the portal and finds a chatbot that uses AI to review all records and offer opinions and perspectives.
Virtual human	A physician diagnoses diabetes after a routine blood count and uses an AI-generated virtual assistant with a human appearance to communicate the diagnosis to the patient via telehealth (without the physician also being present).

a AI: artificial intelligence.

b CT: computed tomography.

### Data Collection

Breakout sessions included small group discussions (2‐4 participants) focused on the specific AI health care scenarios. Each scenario was presented by a dedicated facilitator who rotated between groups, ensuring that all participants discussed all 5 scenarios. Facilitators used standardized, prewritten scripts to introduce each scenario with a concise (approximately 1 minute) verbal description. To ensure consistent understanding, facilitators were prepared to clarify scenario details and answer participant questions as needed, using uniform prompts and clarifications.

For each scenario, facilitators guided the discussion using a structured set of questions designed to explore participants’ perspectives on that specific AI application in a clinical context. The first question asked participants to rate their comfort with the use of AI in the given scenario on a 1‐5 scale (1 being least comfortable and 5 being most comfortable). This question was explicitly framed to focus on the comfort level with the AI application as described in the scenario, not general attitudes toward AI. Additional questions probed what information participants would need to feel confident in the AI’s use, perceived benefits, potential concerns, and preferences for communication of AI-generated results. To promote consistency across discussions, facilitators received training on using the scripts, maintaining neutrality, and applying the structured question guide uniformly. Regular check-ins among facilitators during the session helped ensure alignment in approach and responses to participant questions. Facilitators also encouraged participants to share specific examples and personal experiences to enrich the discussion.

The session was recorded and transcribed verbatim, with facilitator notes collected to supplement the transcripts. A demographic questionnaire captured participant characteristics, including age, gender, health care experience, and self-reported medical conditions. Participants were asked to indicate any chronic illnesses or health conditions as part of a presession survey to better understand how their clinical experiences might inform their perspectives. In addition, participants were asked about their familiarity with AI and the frequency of AI use in their daily lives through structured survey questions, such as “Have you used AI applications like virtual assistants or automated systems? If so, how often?” These responses provided context for interpreting participant perspectives during the session. A postsession evaluation form gathered feedback on the session’s content, structure, and overall experience.

### Data Analysis

Thematic analysis was conducted using an inductive approach to identify patterns and themes within the focus group discussions. Furthermore, 2 researchers (GF and JB) independently coded the transcripts and resolved discrepancies through consensus. The initial coding process involved independently reviewing transcripts and assigning codes that captured key ideas and recurring sentiments. The coding team then iteratively refined and organized these codes into broader themes as a group. The process was informed by the discussion prompts, with themes often reflecting areas of interest, such as trust, communication, and perceived benefits or concerns. However, the themes were not strictly limited to the prompts, as additional insights emerged organically from participant discussions. To ensure the validity and relevance of the findings, the initial themes were presented to a patient-led steering committee as part of our AHRQ-funded Patient-Partnered Diagnostic Center of Excellence. This committee, comprising patient advocates and representatives, reviewed the themes, validated the findings, and provided additional feedback and considerations that were incorporated into the final analysis. Analytical memos documented the rationale for decisions and theme development throughout this iterative process. The final themes were organized to highlight both scenario-specific findings and cross-cutting issues, ensuring a comprehensive understanding of patient perspectives on AI applications in diagnostic communication.

### Ethical Considerations

This study received institutional review board approval by the MedStar Health Research Institute (STUDY00005888), and participation was voluntary. Informed consent was waived under the approved protocol. Participants were provided a US $100 gift card as compensation for their time and contributions during the 2-hour session. All data collected were deidentified prior to analysis to protect participant privacy and confidentiality. No personally identifiable information (PII) was retained or linked to study records. Data were securely stored on password-protected servers accessible only to the research team. These procedures were implemented to ensure compliance with ethical standards for human subjects research, including safeguards for confidentiality and privacy.

## Results

### Participant Demographics

A total of 17 participants attended the AI focus group session, representing a diverse range of perspectives and varied experiences with health care (Table 2).

**Table 2.** Artificial intelligence co-design workshop participant demographics (n=17).

Characteristics	Patients (N=17), n
Age (y)	
18‐24	4
25‐34	4
35‐44	2
45‐54	2
55‐64	2
65‐74	3
75 and older	0
Prefer not to answer	0
Gender	
Men	4
Women	13
Nonbinary	0
Prefer not to say	0
Race	
White (non-Hispanic)	5
White (Hispanic)	0
African American or Black	4
Asian	6
American Indian or Alaska Native	0
Native Hawaiian or Other Pacific Islander	0
More than one race	1
Prefer not to answer	1
Highest level of education	
Some high school	0
High school graduate	0
Some college or associate's degree	2
Bachelor’s degree	3
Master’s degree	10
Doctoral or professional degree	1

Participants reported a variety of medical conditions, reflecting a diverse range of health experiences. These included chronic conditions such as polycystic ovary syndrome, generalized anxiety and depression, hypertension, ulcerative colitis, arthritis, and diabetes. More complex conditions were also represented, such as avascular necrosis, stroke, kidney transplant, heart transplant, cancer, and post-traumatic stress disorder. This range of conditions provided valuable perspectives on the integration of AI in addressing diverse health care needs.

### Patient Comfort Across Scenarios

**Figure 1.**
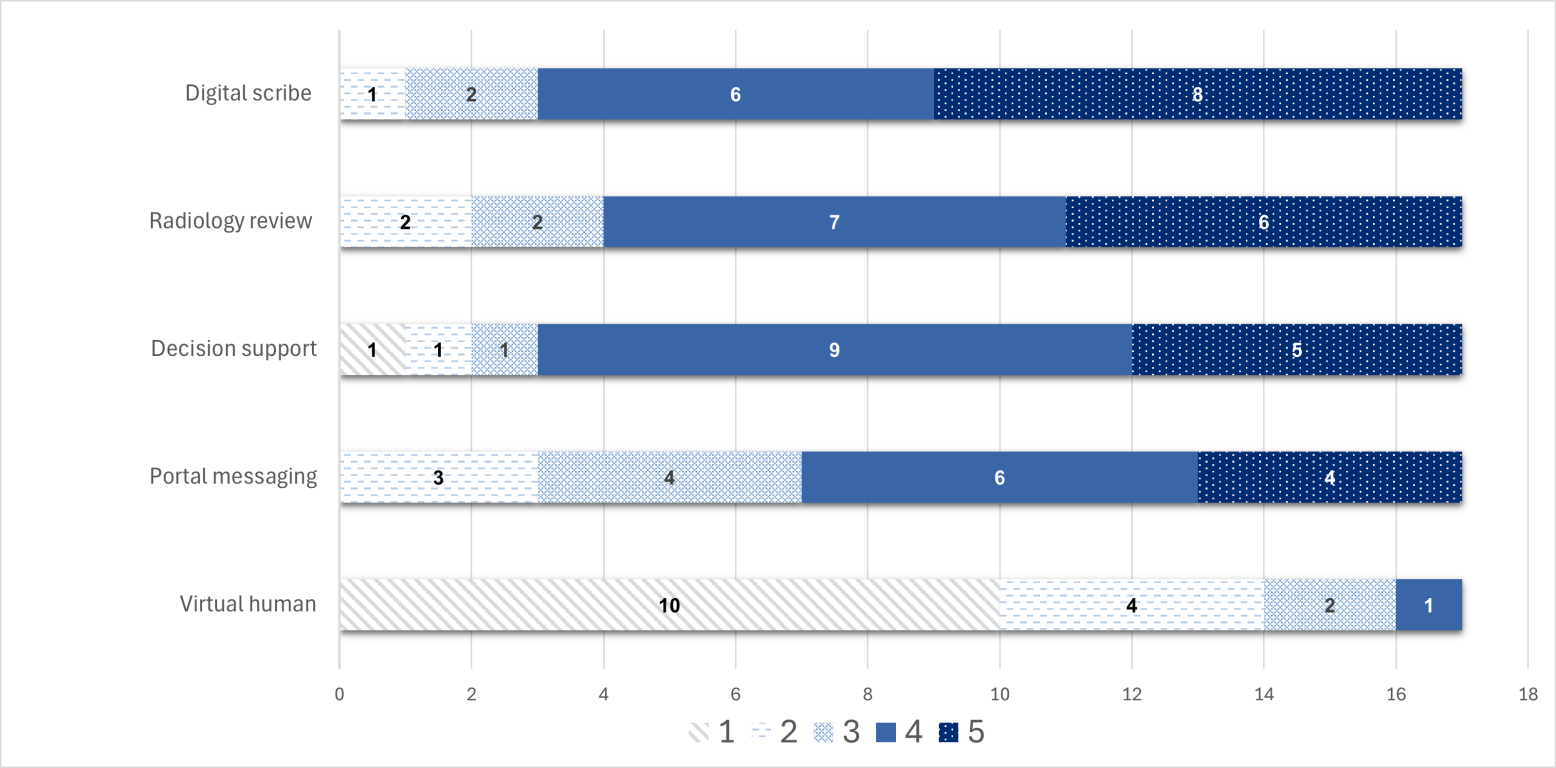
Frequency of participants’ comfort levels with each artificial intelligence scenario on a scale of 1 to 5 (1 being the least comfortable and 5 being the most comfortable).

Participants expressed overall comfort with AI being integrated into the diagnostic process, as long as implementation involved key themes that addressed their concerns and expectations (Table 3, Figure 1). However, participants’ comfort levels varied significantly depending on the level of human interaction involved in the AI scenario. The results showed that comfort level drastically decreased as the amount of human interaction decreased in the AI process. For example, the scenario with which participants were least comfortable was the virtual human telehealth visit in which an AI-generated human would replace the physician when communicating a new diagnosis. Similarly, participants also appeared less comfortable with an AI chatbot sharing details about laboratory results. In contrast, participants were most comfortable with the ambient digital scribe scenario, in which an AI scribe documents clinical notes during a patient visit.

**Table 3.** Participants’ comfort levels (average and range) with each artificial intelligence scenario on a scale of 1 to 5 (1 being the least comfortable and 5 being the most comfortable).

Scenario	Average comfort level, mean (range)	
Digital scribe	4.24 (2‐5)	
Radiology review	4.00 (2‐5)	
Decision support	3.94 (1‐5)	
Portal messages	3.68 (2‐5)	
Virtual human	1.68 (1‐4)	

### Scenario-Specific Findings

Across scenarios, participants expressed a mix of enthusiasm and caution, highlighting specific concerns around AI’s accuracy, transparency, and ability to meet individual patient needs.

#### Digital Scribe

Participants viewed the digital scribe scenario as a promising tool to improve documentation efficiency and reduce administrative burdens on clinicians. However, concerns centered on informed consent and the security, storage, and transfer of data generated by the scribe. Participants indicated a strong preference for receiving notification about the use of an AI scribe before the encounter, with 1 participant expressing:

*I would rather have the opportunity to know about it, think about it, review it, know what the process is, then decide.*

Many participants also questioned how their sensitive health information would be handled, with 1 asking:

*How is the database encrypted? Are you using software that other people won’t have access to? How are we protecting that personal health information?*

Similarly, others sought clarity on how notes were processed and uploaded into their electronic health records, with 1 participant requesting:

*I would like to review it before it gets uploaded...at what point does it get into my chart?*

These concerns reflect a broader apprehension about losing control over personal data in health care settings. Participants were also wary of the scribe’s potential to misinterpret or omit critical details during documentation. They expressed a preference for providers to review and validate the scribe’s work to ensure accuracy and context. For example, 1 participant remarked:

*I think it’s important for my doctor to verify what’s documented—AI might miss something I said.*

Despite these concerns, some participants noted that AI might handle routine documentation tasks more effectively than humans, particularly in scenarios with a high cognitive load for clinicians.

#### Radiology Review

Participants expressed mixed reactions to the use of AI in interpreting radiological images. While many appreciated the potential for AI to identify abnormalities more efficiently and with fewer errors, they also stressed the importance of transparency about the tool’s error rates and limitations. One participant stated:

*For me, I want to know some stats. I want numbers. So percentages in terms of its accuracy, and frequency use…*

Concerns about bias in AI training datasets were also prevalent, with participants questioning whether the tool was designed to account for variations in patient demographics. One participant remarked:

*I would worry about the biases in selecting the populations for these diagnoses. What may look normal for some people could be different for others.*

Many participants agreed that AI could be a valuable supplementary tool for radiologists, but not a replacement. One participant summarized this sentiment, saying:

*If it’s used as a tool by a physician, and the physician is still very much involved, I’m okay with it.*

#### Decision Support

The decision support scenario elicited significant discussion about trust in AI-generated recommendations. Participants were particularly concerned about whether the tool’s algorithms adhered to current clinical guidelines and standards of care. One participant stated, “I’d want to know if it’s based on current standards of care,” emphasizing the need for evidence-based systems. Transparency about how the tool generated its recommendations was also a priority, with one participant asking:

*How did it make that decision? What [the AI] is drawing its information from truly makes a difference.*

Participants highlighted the importance of maintaining provider oversight in decision-making, expressing discomfort with the idea of AI functioning autonomously. One participant remarked:

*I don’t want the AI to be the final say for my diagnosis. I think the doctor should have that final say.*

However, some saw value in AI serving as a secondary layer of support, particularly for routine or low-stakes tasks, such as flagging potential issues in laboratory results or medical records.

#### Portal Messages

The portal messaging scenario was met with cautious optimism. Participants valued AI’s ability to summarize test results and provide routine reminders but raised concerns about its ability to personalize messages. One participant questioned:

*If we all use the same algorithm, but we have different diet habits or lifestyles, how does it account for those differences?*

Transparency and communication were critical to participants’ comfort with this scenario. They emphasized the need to clearly distinguish between AI-generated messages and clinician-written notes. One participant stated:

*I want to know upfront if this is summarizing or interpreting my results.*

While participants generally supported the use of AI for straightforward tasks, they were less comfortable with it providing interpretations or clinical recommendations without a provider’s input.

#### Virtual Human

The virtual human scenario sparked significant debate about the appropriateness of AI for certain types of interactions. Participants expressed openness to using AI for follow-up care or routine questions, such as those related to medication instructions or dietary advice. One participant noted:

*I think it would be a good use as a supplement...If you forget something from the doctor’s visit, you could go back and use the AI for that purpose.*

However, participants were clear that AI should not replace human clinicians in delivering sensitive or high-stakes information, such as a serious diagnosis. One participant stated:

*If you’re telling me I have a brain tumor, I don’t want AI telling me that.*

Others emphasized the importance of empathy and understanding, which they felt AI systems could not replicate. For example, one participant shared:

*When it comes to major lifestyle issues, I’d rather hear personally from my doctor to get some empathy and understanding.*

### Cross-Cutting Themes

**Figure 2.**
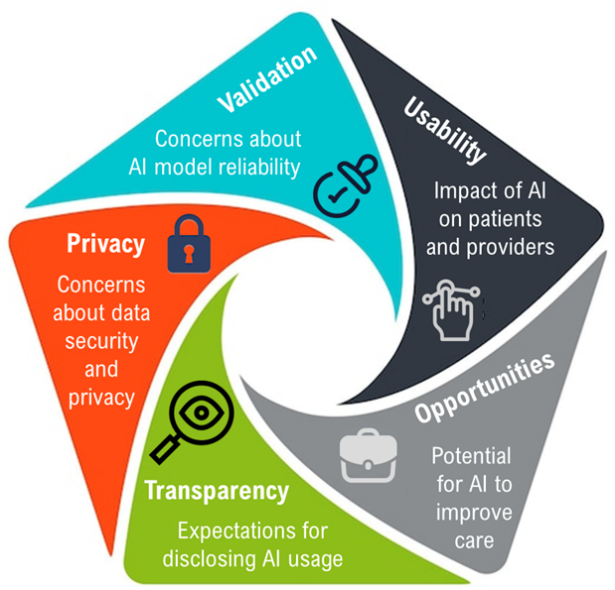
Key themes identified from participant discussions, highlighting concerns, expectations, and opportunities related to the integration of AI into health care. AI: artificial intelligence.

Analysis of the co-design session discussions revealed 5 key themes that highlighted participants’ concerns, expectations, and opportunities for AI integration into clinical workflows: validation, usability, opportunities, transparency, and privacy (Figure 2). These themes provide critical insights into how patients perceive and evaluate AI technologies, which were further reflected in their comfort levels across different AI implementation scenarios.

#### Theme 1: Validation—Concerns Around Model Development and Accuracy

Participants emphasized that trust in AI tools hinges on their validation through rigorous processes to ensure safety, accuracy, and reliability. Across scenarios, they raised questions about how AI systems are developed, trained, and evaluated to meet clinical standards. Many participants expressed a desire for transparency about the data sources used to train AI models and whether these systems could handle the complexities of health care. One participant in the decision support scenario asked:

*Where does this data come from that it’s following algorithms? What features is it using?*

Similarly, in the digital scribe scenario, another participant queried:

*What’s its database? And where’s it pulling its information from to make translations as a scribe?*

Concerns about accuracy and reliability were prevalent, with participants wanting clarity on error rates and diagnostic limitations. These comments highlight a strong preference for metrics and transparency regarding AI performance. Participants also emphasized that AI tools should be aligned with clinical guidelines and standards of care to ensure they provide evidence-based recommendations. Without robust validation processes and clear communication regarding reliability, participants expressed skepticism about trusting AI systems.

#### Theme 2: Usability in Supporting Diagnostic Processes and Communication

Participants discussed AI’s role in achieving effectiveness, efficiency, and satisfaction in diagnostic processes and communication. They consistently emphasized that AI tools should act as supportive, assistive technologies that enhance provider workflows and patient experiences, rather than replacing human decision-making or interactions. Across scenarios, participants expressed a strong preference for AI to serve as a secondary tool that complements clinician expertise, ensuring accuracy and maintaining trust. For example, 1 participant in the virtual human scenario stated:

*I think it would be good to use as a supplement or a good reference point...but I don’t think it should be used as the primary source of education for any diagnosis.*

This reflects a concern for maintaining human oversight and judgment in critical health care decisions. Participants also highlighted the importance of tailoring AI’s involvement to the complexity and context of the task. Many were more comfortable with AI handling routine or low-stakes tasks, such as summarizing medical records or flagging potential issues, as these functions contribute to efficiency without undermining patient-clinician communication. In the decision support scenario, 1 participant noted:

*Routine testing I would feel comfortable for, like diabetes or things like blood pressure.*

However, they expressed hesitancy about AI’s ability to independently manage complex or high-stakes decisions, where human expertise is essential. While participants appreciated AI’s potential to improve efficiency, they consistently emphasized that these gains should not come at the cost of quality, personalization, or the human connection in care. Balancing these considerations is essential to ensure that AI tools achieve their intended usability in the diagnostic process and communication.

#### Theme 3: Transparency—Expectations Around Disclosure of AI Usage

Transparency emerged as a critical factor for fostering trust in AI tools. Participants consistently emphasized the need to understand AI’s role in their care, its capabilities, and its limitations. Across scenarios, participants requested clear explanations of what contributions AI made to clinical decisions or communication. In the radiology review scenario, 1 participant stated:

*If the AI found something, I would want to know if it was found by the AI.*

Another participant in the digital scribe scenario expressed a similar sentiment, asking:

*How is this being processed and what is the accuracy?*

Timing of communication also mattered to participants, with many stressing that AI usage should be disclosed before it is implemented in their care. In the portal messaging scenario, 1 participant said:

*I want to know about it [AI involvement] before I get to the doctor...I’d rather have the opportunity to think about it and review it beforehand.*

Informed consent for AI usage was particularly important in high-stakes situations, with participants calling for providers to explain AI’s role and limitations clearly. Transparency, participants felt, was not just about disclosure but also about respecting patient autonomy and ensuring they have the necessary information to make informed decisions.

Discussions in the radiology review scenario also reflected concerns about how AI tools integrate into clinical workflows. Participants wanted assurances that these tools enhance rather than disrupt existing systems, emphasizing the importance of effective implementation strategies that support both patients and providers. One participant queried:

*Are they going to connect it to the machine that’s doing the scans or the MRIs?*

Such concerns highlight the need for clear communication about how AI integrates into care processes to maximize effectiveness and satisfaction for users.

#### Theme 4: Opportunities—Excitement and Opportunities for AI to Better Address Patient Needs

Despite their concerns, participants expressed optimism about AI’s potential to improve patient engagement, understanding, and comfort. Many viewed AI as a valuable tool for providing supplemental information, clarifying complex medical concepts, and answering follow-up questions. In the virtual human scenario, one participant remarked:

*I like the idea of having supplemental information I can access outside of appointments.*

In the portal messaging scenario, another participant noted:

*I would say I like the idea of having the supplemental information and being able to access that.*

Participants also highlighted AI’s potential to enhance comprehension for patients with limited health literacy or digital skills. One participant shared:

*My parents, when they read their medical history and the doctor’s notes, have no clue what any of it means. They have to put it in ChatGPT so it could be easier to understand.*

However, participants emphasized that the effectiveness of AI in these roles depends on its accessibility, adaptability to diverse patient populations, and ability to integrate seamlessly into existing systems. Barriers, such as digital literacy gaps and language differences, were flagged as critical considerations for designing inclusive AI tools.

#### Theme 5: Privacy—Patient Concerns Around Data Protection, Privacy, and Security

Concerns about data privacy and security were prominent across all scenarios, with participants expressing apprehension about how their sensitive health information would be stored, accessed, and used. Participants in the digital scribe scenario were particularly concerned about data transfer and storage, with one asking:

*Is it transferable? Is it something that would stay within my healthcare unit, or could others access it?*

Another participant queried:

*How is it stored? Is it going to be posted on the patient portal? Are we able to access it?*

Participants also raised concerns about potential misuse of data, particularly for non–care-related purposes. In the virtual human scenario, 1 participant worried about the possibility of data mining, asking:

*Would it be a gain for me, like if it was mining my data?*

Similarly, in the portal messaging scenario, a participant asked:

*How much does the chatbot know?*

These concerns underscore a broader mistrust of data handling practices and the need for robust privacy protocols to safeguard patient information. Participants consistently called for systems to prioritize transparency and informed consent regarding data collection and usage, ensuring that personal information is used solely for its intended purposes.

### Participant Evaluation of the Co-Design Sessions

Participants provided largely positive evaluations of the co-design session (Table 4).

**Table 4.** Participant evaluation of the artificial intelligence (AI) co-design workshop (N=17).

Overall experience	Response, n (%)				
	1	2	3	4	5
How would you rate the meeting overall?^a^	0 (0)	0 (0)	0 (0)	2 (12)	15 (88)
In general, how useful was the meeting?^b^	0 (0)	0 (0)	0 (0)	4 (24)	13 (77)
What did you think about the materials presented and discussed during the meeting?^b^	0 (0)	0 (0)	0 (0)	5 (29)	12 (71)
What did you think about the guideline/recommendation discussion?^b^	0 (0)	0 (0)	1 (7)	5 (33)	9 (60)
How much did the meeting contribute to a shared awareness of AI and diagnostic safety?^c^	0 (0)	0 (0)	3 (6)	8 (47)	8 (47)
How much say did you feel you had in the discussion?^d^	0 (0)	0 (0)	2 (12)	0 (0)	15 (88)
Do you think that the opinions of the different stakeholders that were present at the meeting were all taken into consideration?^d^	0 (0)	1 (6)	3 (18)	6 (35)	7 (41)

a 1=Poor, 5=Excellent.

b 1=Not useful, 5=Very useful.

c 1=Almost nothing, 5=A lot.

d 1=Not at all, 5=Very much.

Participants highlighted several aspects of the workshop that they liked best. They appreciated the open and nonjudgmental environment, which allowed for free sharing of opinions and thoughts without bias or pressure. Many valued the interactive nature of the session, particularly the small group discussions, which facilitated deeper engagement, diverse perspectives, and meaningful participation. The diverse backgrounds of participants, including patients from different races and professions, enriched the discussions and provided new insights. Participants also found the materials well-prepared, appreciated the brief AI introduction, and enjoyed the opportunity to learn more about AI in relation to their health care. Overall, the combination of open dialogue, group interaction, and thoughtful organization was highly praised.

Participants shared a few areas for improvement in the workshop. The most common concern was the limited time available, with several noting the need for more time to discuss topics in greater depth and brainstorm ideas. Some also suggested dedicating additional time for group discussions and addressing specific examples of AI currently in use or relevant case studies. Suggestions included incorporating more complex cases or scenarios, discussing AI bias in greater detail, and diversifying both the researcher backgrounds and participant groups to include more primary care providers, individuals from different socioeconomic groups, and a broader generational representation. While some recommended separating patients based on their AI knowledge for tailored discussions, others emphasized maintaining a mix of diverse perspectives within groups. Overall, participants highlighted opportunities to enhance inclusivity, depth of discussion, and time for meaningful engagement.

## Discussion

### Principal Results

AI, while previously a technology of the future, has become a technology of the present. AI-driven technologies, including machine learning-driven decision support algorithms, deep-learning radiology scan classifiers, and large language model–driven digital scribes, have already been implemented in hundreds of hospitals nationwide. This study, in examining patient perceptions of 5 different scenarios describing current and future AI technologies in health care, provides a contemporary view of the multifaceted patient perspectives on AI’s role in providing diagnostic information, facilitating communication, and supporting decision-making. Many patient perceptions held true across all scenarios. First, a need for transparency in the development and validation of AI models, including their ability to reliably address the diverse needs of patients. Second, a preference for AI to complement rather than replace human providers, with an emphasis on maintaining human oversight in clinical decision-making. Third, the importance of clear and respectful communication about AI’s role in care, including obtaining patient consent, was seen as essential for building trust. Fourth, the potential for AI to enhance patient engagement, understanding, and access to information, provided it is implemented as a supportive tool that respects patient autonomy. Finally, concerns regarding the security and privacy of patient data highlight the need for transparency and robust safeguards to prevent misuse or unauthorized access.

The outlook on AI implementation into the diagnostic process was generally positive, with participants consistently highlighting AI’s ability to identify patterns and provide supplemental diagnostic information that might otherwise be overlooked by human providers. However, they emphasized that AI tools must integrate seamlessly into clinical workflows and preserve the essential human connection in patient-provider communication, as seen in the results regarding patient comfort levels across scenarios. Participants reported lower average comfort levels with high AI-patient interaction scenarios. For example, the scenario participants were least comfortable with was the virtual human scenario in which an AI-generated human would replace the physician when communicating a new diagnosis. Similarly, participants also appeared less comfortable with an AI chatbot sharing details about laboratory results. In contrast, participants were most comfortable with the digital scribe scenario, in which the application of AI was intended to enhance patient-provider communication by removing the need for providers to focus on documentation during clinic visits.

These findings highlight the importance of understanding patient perspectives within their specific health care contexts, offering insights into how AI can be integrated to enhance diagnostic processes and communication. For example, rural communities, where health care access and infrastructure often differ from urban settings, may present unique opportunities for AI to address gaps in care. Designing AI systems that are adaptable to varying levels of digital literacy, resource availability, and cultural expectations can help ensure these tools are both effective and equitable across diverse populations. Similarly, the interactive focus group discussions demonstrated the value of engaging patients with varying experiences and levels of familiarity with health care technologies. This diversity of input underscores the potential for AI to be developed in ways that resonate with patients from different backgrounds, fostering trust and satisfaction. By actively seeking and incorporating a wide range of perspectives, AI tools can be tailored to address the specific needs of different communities, ultimately supporting a more inclusive and patient-centered approach to care.

### Comparison With Previous Work

The findings of our work that held true across all scenarios are expectedly consistent with previous work – patients’ concerns with privacy, data security, and bias have been well-documented [26,27]. Specifically, our findings align well with the findings that patients have a generally positive outlook on AI’s implementation into their care as long as there are adequate guardrails to protect against a variety of potential harms [28]. Our work was unique in its focus on how such concerns are explicitly perceived in the context of AI applied to diagnosis and its communication in a variety of clinical contexts. Few qualitative studies have explored patient perceptions of AI in the context of diagnosis and communication, but our results appear to be in alignment with previous findings. Patients considered AI to be a helpful supplementary tool that should not serve as a replacement to human clinicians, a sentiment already documented for applications of AI in radiology [23].

While there is previous work identifying patient perceptions on the implementation of AI in health care broadly, there has been limited work identifying patient perceptions of AI’s role in reducing diagnostic errors through the enhancement of patient-provider communication. A recent scoping review identified that patients’ attitudes toward AI (which may impact their experiences when they interact with these tools in practice) are influenced by various factors, including familiarity with function, previous exposure to similar tools, supervision during use, and tool simplicity, validity, and cost. In light of this, it is imperative to consider patient perceptions of AI applications in a variety of clinical workflows in the context of diagnostic communication [17]. As AI becomes ubiquitous both inside and outside of health care, patients’ familiarity (and thus their attitudes toward AI) will continue to evolve. It is critical that patient perceptions of AI tools are continually assessed and used to enhance the diagnostic process and communication so that AI can be designed and integrated into the health care system in such a way that maximizes patient care and satisfaction.

### Implications at the Individual Clinician, Organizational, and Policy or Regulatory Levels

Our findings have clear implications for clinical practice, finding that patients consistently stressed the importance of clinicians playing a central role in facilitating their experience with AI tools. Patients emphasized that their trust in AI would be built through transparent communication and clinician endorsement. Patients valued clinician involvement in contextualizing AI’s outputs, interpreting its recommendations, and providing assurances about its accuracy and reliability. Patients additionally expressed concerns that AI could disrupt workflows or reduce human interaction, particularly in scenarios where key diagnostic information was to be communicated. To address this, organizations should prioritize the implementation of AI tools that enhance human connection in care, such as those that reduce administrative burdens. These tools should be designed in ways that enhance, rather than overshadow, the clinician’s role in communicating diagnoses.

From a policy perspective, our findings reinforce the urgency of addressing gaps in regulations governing AI in health care, particularly concerning equity [29]. Patients expressed concern that biased data inputs could undermine the diagnostic accuracy of AI tools and have harmful effects on historically underserved populations. Policies should ensure that tools are trained on diverse datasets and are validated across representative populations in order to build patient trust and acceptance. Patients also expressed calls for transparency and informed consent regarding how health data is collected and used by AI systems in health care, aligning well with findings from a study [30]. These concerns should be addressed by policies that establish clear standards for disclosing when AI is used in care, and for ensuring that patients understand what personal information is being used, how it contributes to the diagnostic process, and how it influences the outputs from AI systems. Strengthening privacy and communication protocols will not only help address these concerns but also reinforce patient autonomy and trust in AI by supporting its ethical implementation in clinical settings.

### Importance of Involving Patients in AI Deployment

Our findings underscore the critical role patients play in the acceptance and success of AI tools designed to enhance the diagnostic process, emphasizing the need to involve them in the development and implementation of these technologies. As primary stakeholders most directly impacted by changes to diagnostic workflows, patients have invaluable insights that can guide the design of tools to align with their expectations and foster trust [31]. Participants in this study expressed a dynamic view of AI tool implementation, with key insights into concerns that should be addressed during design and implementation, such as the importance of human connection and interaction, concerns regarding equity, personalization, and data security, and the pivotal role clinicians have in their understanding and comfort with new technology. By involving patients in the development of these tools, the health care system can better anticipate risks, communicate with patients more effectively, and deploy tools that not only improve the diagnostic process but also enhance trust and adoption, ensuring alignment with patient values and priorities.

### Limitations

This study has several limitations. First, focus groups were guided by different facilitators, which may have introduced variability in discussions due to differences in facilitation styles. The breakout sessions included only 2-4 participants per group, which allowed for in-depth discussions but may have limited the diversity of viewpoints within each session. This could affect the robustness of the findings; however, insights were aggregated across groups to capture broader themes. Future studies could address this by increasing group sizes or incorporating complementary methods such as individual interviews. Time constraints limited the discussion duration for each scenario, potentially restricting exploration of nuanced perspectives and the ability to achieve thematic saturation. In addition, the use of specific diagnostic-related AI scenarios provides structure but limits the generalizability of findings to other clinical contexts.

Participants were recruited through advisory networks, which may have introduced selection bias favoring individuals with an interest or familiarity in health care technologies. The participant demographics also reflect limitations in diversity, as more than 70% (n=13) were women, almost 65% (n=11) held graduate degrees, and none identified as having a Hispanic background. In addition, no participants reported high school or lower as their highest education level. According to national data, approximately 62% (n=115,011) of individuals aged 25 years and older in the United States have not attained a bachelor’s degree, suggesting that our sample overrepresented highly educated individuals [32]. Perspectives from participants with less formal education or from underrepresented backgrounds may have differed significantly, potentially revealing lower trust in AI, different concerns about its use, or alternate expectations for its role in health care. This lack of demographic diversity may limit the generalizability of the findings, as perspectives from individuals with different educational or cultural backgrounds could provide unique insights into AI applications in health care. We also did not assess baseline AI familiarity or digital literacy, which may have influenced participant engagement. Finally, this study is not comprehensive of all patient concerns about AI, with a focus on diagnostic applications shaping the discussions. Future research should aim to include a more demographically representative sample, explicitly assess AI literacy, and explore a wider range of clinical and nonclinical AI use cases to better understand how diverse patient populations perceive and respond to its implementation in the health care setting.

### Conclusions

This study highlights the nuanced perspectives of patients on the use of AI in health care, with a particular focus on diagnostic communication. While participants recognized the potential of AI to improve diagnostic accuracy, efficiency, and equity, they also voiced significant concerns about transparency, trust, and the preservation of human connection. These findings underscore the importance of ensuring that AI tools are developed and integrated in ways that align with patient values and priorities. Key patient-oriented considerations include the need for clear communication about AI’s role in care, consent processes for its use, and opportunities for patients to actively participate in its development and implementation. Participants emphasized the importance of maintaining provider oversight, fostering understanding through accessible explanations, and designing systems that prioritize inclusivity and respect for patient autonomy.

As AI technologies continue to evolve and permeate health care, it is essential to iteratively assess and incorporate patient feedback to ensure these tools not only meet technical and clinical standards but also uphold the values of equity, transparency, and shared decision-making. By centering patients in the design and deployment of AI, we can create systems that not only enhance health care delivery but also foster trust and meaningful engagement between patients and providers.

## Supplementary material

10.2196/69564 Multimedia Appendix 1 Artificial intelligence (AI) scenarios for breakout discussion.

## Abbreviations

AI: artificial intelligence
