# Supplementary material for: Patient Perspectives on Artificial Intelligence in Health Care: Focus Group Study for Diagnostic Communication and Tool Implementation
Source: J Particip Med. 2025 Jul 24;17:e69564. doi: 10.2196/69564 (PMC12288699; doi:10.2196/69564)
Supplement: Multimedia Appendix 1 [file jopm-v17-e69564-s001.docx]

# Appendix 1. AI Scenarios for Breakout Discussion

**Use of AI for Patient Portal Messaging (Portal Messages):** You finish a clinical visit with your physician about a health concern, and he recommends lab work to rule out several different potential diagnoses. You forget to take notes during your appointment and log into the patient portal to review your providers’ notes and lab results. In reviewing the patient portal, you see that you have an option to ask a ChatBot questions about your appointment and results. The ChatBot tells you that an artificial intelligence system will interpret your medical history, notes, and results, and share its opinion and perspectives.

**Use of AI in Radiological Imaging Review (Radiology Review):** Imagine you visit your primary care provider complaining of persistent lower back pain which has been making it difficult for you to perform daily activities. Concerned about the severity of your symptoms, your physician recommends a series of diagnostic tests including a radiology scan, to determine the cause of your plain. The radiologist doesn’t see anything concerning but when an AI software is applied that reviews the image, it identifies a herniated disc pressing on your spinal nerve. The radiologist concerns the diagnosis and reports back the finding to your primary care physician.

**Ambient Digital Scribe (Digital Scribe):** Imagine a scenario in which you go to see your usual primary care doctor for a common checkup. Per usual, you get assigned to a waiting room and bide your time until the doctor finally enters the room. Before beginning the appointment, the doctor pulls out their phone and says “I would like to use an artificial intelligence-based app on my phone to document our visit. It will listen to our conversation and write down everything that I otherwise would. Do I have your permission to use this AI app?” ***Follow-up Scenario:*** Your doctor continues with the following: “I understand that you may have concerns – but I have been using this AI app for three months with no problems. It is a great help to me, and all recordings are stored in a HIPAA complaint, secure, cloud, so there are minimal security risks.”

**Virtual Human Telehealth (Virtual Human):** You go to your primary care provider to perform a routine wellness visit. A complete blood count is ordered and completed. Upon review of your results, your physician determines that you have a diagnosis of diabetes. To communicate the diagnosis, your provider generates a virtual AI-generated human with the appearance of a physician. The provider invites you to a telehealth visit on behalf of the AI-generated physician. During your telehealth visit, the AI-generated physician communicates your blood test results and associated diagnosis with you.

**Use of AI for Clinical Decision Support – HIV Testing (Decision Support):** During a routine wellness visit, your primary care physician reviews standard information related to your health, past medical history, medications, and social history. As part of your social history, your clinician asks you routine questions regarding your sexual history, including your partners, practices, and prevention tools. They also ask about your use of non-prescribed substances including marijuana, cocaine, and opioids. At the conclusion of your visit, your clinician offers you a screening blood test for HIV. The clinician informs you that screening for HIV is routine in clinical practice and is sometimes recommended for persons depending on various factors. The clinician informs you that an artificial intelligence system interpreted your medical history and created a clinical decision reminder for them that recommended you receive screening.
